# Understanding host-microbial interactions in rumen: searching the best opportunity for microbiota manipulation

**DOI:** 10.1186/s40104-016-0135-3

**Published:** 2017-01-19

**Authors:** Nilusha Malmuthuge, Le Luo Guan

**Affiliations:** 1 Department of Agricultural, Food and Nutritional Science, University of Alberta, Edmonton, T6G 2P5 AB Canada; 2 4–16 F, Agriculture/Forestry Center, Department of Agricultural, Food and Nutritional Science, University of Alberta, Edmonton, T6G 2P5 AB Canada

**Keywords:** Host-microbial interaction, Rumen microbial manipulation, Ruminants

## Abstract

Ruminants utilize a wide variety of dietary substrates that are not digestible by the mammals, through microbial fermentation taking place in the rumen. Recent advanced molecular based approaches have allowed the characterization of rumen microbiota and its compositional changes under various treatment conditions. However, the knowledge is still limited on the impacts of variations in the rumen microbiota on host biology and function. This review summarizes the information to date on host-microbial interactions in the rumen and how we can apply such information to seek the opportunities to enhance the animal performance through manipulating the rumen function.

## Background

Ruminants play an important role in meeting the current and growing demand for meat and milk consumed by human. With the world population reaching 9.2 billion by 2050 [1], sustainable ruminant livestock farming has been suggested as a mean to utilize available feed resources within a system to minimize the use of human-edible grains [2]. However, this may conflict with achieving the growing demand, unless proper techniques are developed and implemented to improve the rumen fermentation. It is known that ruminants utilize a wide variety of dietary substrates that are not digestible by the mammals, through microbial fermentation taking place primarily in the rumen. The rumen is the fore-stomach of ruminants, which harbors highly dense and diverse microbial population. Rumen is generally believed to be functioning with solid feed intake and it is physically and functionally different in pre-ruminants compared to that of adult ruminants until the development of the rumen to carry out microbial fermentation [3].

The rumen microbial fermentation is crucial for the growth and production of ruminants. Thus, the rumen microbial composition and function as well as factors affecting the rumen microbiome (composition and functions), such as diet, age, geographic location, and host species have been well studied in ruminant livestock species. Studies on the rumen microbiota and microbial fermentation process go back to mid 1800s [4]. Despite the long history of studies on the rumen microbiota, attempts to manipulate the rumen fermentation are still producing only short-term results [5, 6]. Adult rumen microbiota is resistant to perturbations and original composition is restored following an intervention with exogenous rumen microbiota and diet [5], suggesting that the microbial manipulation methods are less effective on adult ruminants. Recent studies that focused on early life gut microbiota and its long-term impacts on human health and growth [7, 8] suggest a potential to manipulate microbiota through early life to obtain beneficial effects during adult life. Indeed, dietary interventions on pre-ruminant rumen microbiota have been successful in achieving fairly persistent and long-term results [9–11]. However, the knowledge is still limited on the impact of early interventions on adult production.

The present review compiles existing knowledge on the rumen microbiome to understand the host-microbial interactions in this microbially diverse environment and to identify gap in knowledge that may be necessary in designing effective manipulation methods to improve microbial fermentation in the rumen.

## Host-microbial interactions in rumen

Host-microbial interactions are well defined in the gastrointestinal tract of human, due to their enormous impact on host health. Although metabolic dysfunction and health of the rumen (acidosis and subacute acidosis) are also linked to the composition and functions of the rumen microbiota [12–14], host-microbial interactions in the rumen have mainly been studied to maximize the production performance of ruminant livestock species. Moreover, the majority of studies on host-microbial interactions in the rumen are based on adult animals, reporting associations between the rumen microbiota and feed efficiency in beef [15–17] and dairy [18, 19] cattle, methane emission in cattle and sheep [20–22], milk production in dairy cows [18, 19] as well as the susceptibility to subacute acidosis in beef [23] and dairy [24]. Either the presence/absence of microbial phylotypes or the abundance/density of identified microbial phylotypes is associated with the above-mentioned traits, suggesting the selective increase or decrease of those microbial groups through interventions could potentially improve the related host traits.

Manipulation of the rumen microbiota with the aim to alter the host performance has mainly been done in the adult ruminants through dietary intervention in the past [5]. However, the low efficacy of microbial manipulation strategies on adult ruminants has resulted in an increasing interest in understanding the host-microbial interactions in young ruminants [6]. Consequently, there are increasing numbers of studies to explore the impact of early rumen microbiota on the metabolism of rumen [25], production of methane [9, 26] and expression of host microRNAs [27]. Dietary interventions in goat kids (from birth to 3-month-old) have been shown to alter the microbial composition and influence the host phenotypes (methane emission, volatile fatty acids production) in post-weaning [9–11]. These changes obtained via early interventions lasted for 3 months following the cessation of the dietary intervention [9]. Restriction of protozoa acquisition during the early life of lambs has also been reported to change the rumen microbial composition and fermentation as well as urine metabolites, later in life [25]. Therefore, these studies suggest that alterations in the early rumen microbiota may influence the microbial succession process and the host phenotype. This further indicates that microbial manipulations during the early life, before the establishment of a stable microbiome, may be more effective than the manipulations designed to be used for adult ruminants. The impact of microbial changes on pre-ruminant calves is still very limited and the above studies are mainly focused on lambs or goat kids. It is notable that the rumen microbial composition and their abundances can be varied depending on the host species [28], indicating that similar microbial manipulation techniques may not be applicable to other ruminant species such as cattle. Therefore, there it is necessary to understand host-microbial interactions in depth during the early life to design effective manipulation tools and techniques that may have long-term impacts on different ruminant species.

Although the impacts of microbial manipulation methods have mainly been studied in content- or fluid-associated rumen microbiota, the rumen epithelial tissue-attached bacteria known as epimural bacteria, maintain close associations with the host [29]. Recent studies have reported that the epimural community is more diverse than content-associated community [30] and diet can alter its composition [31]. Moreover, the changes in the epimural bacterial density are associated with host gene expression [23] and ruminal acidosis [32]. Although their functional roles are speculated to be involved in oxygen scavenging, urea recycling [33], the understanding of this population is very limited. Thus, future understanding of host-microbial interactions should take the epimural microbial population into account.

## Impact of host on the rumen microbiota

As described above, researches on host-microbial interactions in the rumen are mainly focused on microbial aspect. The limited knowledge on the role of host in regulating microbiota may prevent the consistency when the same intervention method applied across species as well as for a herd. Furthermore, restoration of original status in the rumen microbial composition, following an intervention, can be varied depending on individual animals. Exchanging of the rumen contents between two dairy cows has shown that some animals restore the originality faster than others, whereas some animals may acquire a slightly different microbial composition from its original [34]. These observations suggest that restoration of the rumen microbial composition is also influenced by the host. Similarly, the impact of host may also exert during the use of microbial manipulation methods, such as probiotics and direct fed microbials [35]. Therefore, microbial manipulation techniques should also need to address the host mechanisms that may influence the rumen microbiota.

Use of mouse models has demonstrated the impact of host genetics on the establishment of individualized gut microbial composition in mammals [36–38]. Moreover, the heritability of gut microbiota has been linked to human health [39]. These studies suggest that host plays an important role in determining its gut microbiome and these changes in the microbial composition can also be vertically transmitted, which may cause health issues. Unlike mouse models that compared the gut microbiomes of pure-breeds, the studies on the cattle rumen microbiota have used sire information to link host genetics to the rumen microbiota, aiming to select efficient animals in beef cattle [40, 41]. These studies have also suggested the potential heritability of the rumen microbiota in cattle through indirect relationship between sire breeds and the rumen microbial composition of progenies [40]. Moreover, these potentially heritable microbial phylotypes could also be linked to the host phenotypes, such as feed efficiency [40] and methane emission [41] in beef steers. The study by Roehe et al. [41] has reported that methane emission and archaeal abundance were different between sire progeny groups and proposed that microbial gene abundance could be used in the selection of animals for lower methane emission. Methane emission is a greater issue in terms of both global warming and feed efficiency of cattle [42, 43] in which inefficient cattle has been linked to a higher emission of methane [36]. Therefore, selection for efficient cattle can provide the increasing demand for meat in future with limited resources, and can potentially lower the greenhouse gas emission from agriculture sector. In depth knowledge on the host-microbial interactions in the rumen, thus, may allow efficient selecting and breeding of animals with economical- and environmental-friendly traits based on microbial markers. Moreover, microbiota associated with such phenotypes can be used to develop microbial manipulation tools and techniques (feed supplements—probiotics, prebiotics) to improve the rumen fermentation. Heritability of microbiota associated with phenotypic traits is an important factor to be considered during this process, because low heritable microbiota may not be a good marker for selection. However, they can be used for microbial manipulation due to the low impact of host, whereas highly heritable microbiota can be used to accelerate the animal selection process. Therefore, it is necessary to understand the heritability of phenotypic traits-associated microbiota and the impact of rumen microbial manipulations on these heritable microbial groups as well as their interaction with host and other microbes in future.

Besides the host genetics, age and/or growth of host is also a main determinant of the rumen microbial composition [44–47]. However, studying the impact of host age alone has always been difficult in ruminants, especially the pre-ruminants, due to the con-founding effect by the diet. Pre-ruminants, especially dairy calves, undergo from a whole milk-based to a solid-based diet within a short period of time (eg: dairy calves are weaned by 8 weeks of age) to facilitate the early rumen development, and the studies investigating the impact of calf age on the rumen microbiota [46, 47] have used animals that underwent through these dietary regimes. Thus, these studies are reporting the impact of growth (age and dietary changes) on the rumen microbiome, instead of the impact of host age. Although age-dependent variations in the composition of rumen microbiota were often confounded by diet [45–47], Li and colleagues [44] showed significant changes in the microbial composition and functions that were purely associated with calf age. Relative abundance of *Bacteroidetes* increased from the second week to 6^th^ week of life, whereas abundance of *Firmicutes* and *Proteobacteria* decreased with calf age, when calves were fed a milk-replacer diet [44]. Age-dependent variations were more distinctive at lower taxonomic hierarchy in which the abundance of *Prevotella* (phylum *Bacteroidetes*) decreased and the abundance of *Bacteroides* increased in 6-week-old calves compared to 2-week-old calves [44]. However, when the rumen microbiota of milk-replacer fed calves was compared to that of 12-month-old bull calves fed a hay-based diet, Li et al. [44] observed a higher abundance of *Prevotella* in older bull calves. When the same 6-week-old calves were compared with lactating cows fed a total mix ration (corn silage:concentrate - 50:50), it also revealed a lower abundance of *Bacteroides* and higher abundance of *Prevotella* in cows [45]. An increase in the abundance of *Prevotella* with calf growth was also evident when calves were fed milk-replacer and hay along with gradually increasing levels of grain [47]. Moreover, the abundance of *Bacteroides* increased during the first 9 days of life when calves received milk-replacer and hay, but decreased with the introduction of grain into the calf diet [47]. Therefore, these studies imply that age-dependent changes in the microbial composition vary with dietary changes and the impact of host age can be prevailed by diet. Thus, it is important to understand how dietary interventions influence host-microbial interactions in the pre-ruminant rumen, which may provide evidence to identify best window of time and best approach for microbial manipulation.

The rumen microbial composition can also be influenced by the fraction of the rumen samples and distinct microbiota has been identified for the particle-attached, fluid-associated, and tissue-attached fractions of adult cattle rumen [48]. Microbiota from different fractions may have different interactions with the host. Among them, as described previously, the tissue-attached (epimural) microbiota interacts closely with the host than content-associated microbiota due to their close proximity to host tissue [29], which also suggests a greater impact of host on epimural microbial community. Composition of tissue-attached bacterial community in lambs has been shown to change rapidly during the first 3-weeks of life [49]. Whereas, distinct bacterial compositions between tissue-attached and content-associated bacteria were reported in 3-week-old dairy calves [50]. A recent study in goat kids revealed that the composition of the rumen tissue-attached bacteria changes during growth with age and dietary changes [51]. However, the confounding effect of diet in the study carried out by Jiao and colleagues [51] do not allow understanding the impact of host alone on the epimural microbiota. Epimural bacterial community maintains close association with the host. Therefore, it is necessary to understand the age-dependent variations in the host-microbial interactions in the rumen epimura without dietary interventions. This may allow manipulating the epimural-microbiota that has been suggested to play an important role in the absorption of nutrient via sloughing off the keratinized epithelial cells [33]. The epimural bacteria have been reported to change with rapid grain adaptation in beef heifers, a common practice in feedlots [31]. This study utilized the same animals to undergo through the adaptation period; therefore, they had control the impact of host on the rumen microbiota. Yet, the comparison of bacterial densities among individuals has shown that variations in bacterial densities through the dietary changes are different depending on animal [31]. Thus, understanding the impact of host alone on the establishment of this community and how this process can be manipulated via dietary changes is of greater interest. It is evident that the present knowledge regarding the impact of host on the rumen microbiota has always studied under the effects of confounding factors, a hurdle that need to be addressed properly in future.

## Impact of the rumen microbiota on host

Establishment of host-associated microbiome is a two-way interaction that is modulated by host and microbes [52]. Once established, host-associated commensal microbiota plays important roles in host metabolism and health [53]. The rumen microbiota plays a greater role in the metabolism of ruminants via producing 70% of daily energy requirement of host [54], thus, manipulation methods to maximize the microbial fermentation are widely studied in ruminants [55], aiming to increase the available volatile fatty acids for the host and/or to decrease available substrates for methane production. Studies have shown that the rumen microbiota influence cattle feed efficiency through the production of volatile fatty acids [56]. A most recent study has also reported differences in the production of volatile fatty acids by the rumen microbiota in cows with differing feed efficiency [57]. This study further reported that the rumen metagenome (taxonomy and functions) was also different between inefficient and efficient animals. For example, *Megasphaera elsdenii* that produce butyrate from lactate was high in efficient cows; whereas, *Methanobrevibacter ruminantium*, the most abundant methanogenic archaea in the rumen, was high in inefficient cows [57]. These observations indicate that differences in the rumen microbial groups that involve in various fermentation pathways may have contributed to the differences in the production of volatile fatty acids, which eventually impact on host feed efficiency. However, as described in previous section it is suggested that animals with differed feed efficiency/methane emission may have inherited its microbiota from sire [40, 41]. Thus, it is necessary to understand whether the rumen microbiota is either the cause or results of cattle feed efficiency/methane emission. Besides, these studies have also had confounding impact of diet on the host-microbial interactions, which complicate the interpretation of existing data. In a recent study Jami and colleagues [18] reported a linkage between the rumen bacteria and milk composition in dairy cattle. This study revealed that a higher abundance of *Prevotella* was associated with a lower milk yield. *Prevotella* is one of the dominant bacterial groups colonizing the rumen that consists of various species [58] and various metabolic capacities [59]. Moreover, a recent study has shown that the abundance of varying operational taxonomic units (OTUs) belong to genus *Prevotella* has varying associations with milk composition [19]. Therefore, caution should be taken in interpreting the associations between such a complex bacterial genera and host phenotype. Except for few studies trying to link the rumen microbiota directly to the ruminant phenotypes (milk composition, subacute ruminal acidosis, methane emission and feed efficiency), most of the studies evaluated impact of the rumen fermentation on host performance [55] and knowledge is lacking on direct impact of the rumen microbiota on host performance. In addition, linkages between ruminal subacute acidosis and the rumen microbiota are emerging [23, 24, 60, 61]; however, once again causal effects are not clear.

Furthermore, the knowledge on host-microbial interactions during the rumen development is also lacking. Microbial colonization, morphological and metabolic development of the rumen has been studied individually [62]; however, the impact of the early rumen microbiota on the rumen development has yet to be explored. Regardless, manipulation of the early microbial composition in goat kids has been shown to influence the production of volatile fatty acids and methane [9–11], which are also considered as the parameters of the rumen development. Sequencing of the rumen metagenome of 2-week and 6-week-old calves identified glycoside hydrolases, even at the absence of a solid diet [44], indicating the colonization of metabolically active microbiota during the early life. Hence, future studies to investigate the pre-ruminant rumen microbiome may provide in depth knowledge regarding the role of early rumen microbiome on the development of rumen.

## Dietary manipulation of rumen microbiota to improve ruminant livestock production

Diet is a major cause of changes in the rumen microbial composition, which may overpower the effect of host [59]. A global rumen census effort using a large number of geographically diverse rumen samples revealed that a forage-based diet results in a higher abundance of bacterial order *Bacteroidales* and family *Ruminococcaceae*, whereas a grain-based diet results in a higher abundance of genus *Prevotella* and family *Succinivibrionaceae*, regardless of the ruminant species [63]. Thus, the impact of the rumen microbiota on the production of ruminants has mainly been explained using the dietary manipulation-based studies that influence microbial composition. Diet influences the rumen microbial composition within ruminant species as well as within an individual throughout the growth. For example, the rumen microbial composition was different between beef and dairy cattle that received different diets [45]. Moreover, predominant bacterial and methanogen phylotypes changed, when beef steers were switched from a low-energy diet to a high-energy diet [56, 64]. Dietary manipulation in ruminants is aiming to improve the rumen microbial fermentation either via enhancing beneficial metabolic processes or minimizing inefficient/harmful metabolic processes [55]. The existing rumen microbial manipulation methods include ionophores (monensin), direct fed microbials, and probiotics [5]. However, the effects of dietary manipulations on the adult rumen microbiome and the rumen fermentation are short-lived and present only during the treatment period [55], suggesting that dietary manipulations need to be done during a certain window of opportunity that can trigger persistent changes in the microbiome.

The early life rumen (pre-ruminant rumen) microbiome is less diverse and less stable than that of adult microbiota [46]. Recent studies have shown that the rumen microbiota and functions can be altered in goat kids using dietary interventions during pre-weaning period, which can last for 4 months post-weaning [9, 11]. Similarly, another recent study revealed that a dietary intervention in pregnant ewes along with a microbial inoculation of lambs after birth (rumen fluid of ewes fed different diet) could successfully change the rumen microbial composition of lambs [65]. Although the authors claim that the observed changes were due to the ewes’ diet, the variations in the rumen microbiota of lambs might be due to the introduction of different inocula obtained from ewes during the early life. These preliminary results suggest that dietary interventions during the early life may have long-term effect on the rumen function. However, the best window of time for dietary manipulation to have persistent impact on the rumen microbiota and fermentation process is yet to be understood.

## Knowledge gaps and future directions

The studies in ruminant livestock species (cattle, sheep, goat) are aiming to improve the rumen microbial fermentation as a mean to increase livestock production. However, the fundamental understanding of host-microbial interactions and their regulatory mechanisms is lacking. As described in this mini-review, the rumen microbiota has been widely studied using the recently developed culture independent high throughput sequencing; however, there are still many gaps in knowledge to fill. Firstly, microbial phylotypes are mainly characterized based on similarities of 16S rRNA gene sequences to known microbial taxa. For example, OTUs and/or phylotypes have been used as units to assess the variations in the rumen microbial composition. It is, however, not clear whether these identified OTUs/phylotypes are biologically meaningful for their functional aspects or whether they are the results of artificial bioinformatics analyses. Secondly, most of the rumen microbiota analyses only identify the presence or absence of particular microbial phylotypes under different treatment conditions. Yet, how such compositional differences play a role in specific rumen functions are not well characterized. Thirdly, although emerging studies have been able to identify the rumen microbiome at structural and functional levels using metagenomics and/or metatranscriptomics as well as the associations of these changes with host phenotypes, the conclusions are mainly based on gene/transcript level using the commonly accepted statistical analyses. There is no conclusive understanding whether the alteration in microbiota/microbiome causes the host functional changes or whether such changes are the result of host physiological changes, due to the lack of validation model systems to verify their relationship. Recent advanced technology has allowed to identify metabolites of rumen microbiota [27], of which, the changes in the some of them could impact both microbial and host activities. Also, the host physiological and biological changes may also lead to the changes in microbiome and their metabolites. However, the understanding of the rumen microbiome, metabolome and their host, is lacking. Lastly, many studies have used very few numbers of animals and the individualized rumen microbiome has been largely ignored or unaccounted during nutritional trials. Furthermore, many researches related to the rumen microbiota lack of data collection, especially on animal genetic background and environmental features. To date, the linkage of the rumen microbiome to host mechanisms is largely missing. For example, the association of the rumen microbiota with the rumen size, physiological changes, and passage and digestive rate as well as role of host genetics is still a large “black box”. Future collaborative efforts in designing meta-trials to include all information (diet composition, management, host genetics, animal health status, volatile fatty acids production, rumen pH) from various animal trials with comprehensive datasets and new data analysis methods are needed to define the window and the best strategies to manipulate the rumen microbiota. Such collaborative efforts can overcome the limitation caused by the use of few numbers of animals by individual researches and can be used to identify the impact of host genetics on rumen microbiome.

## Conclusion

In summary, the existing microbial manipulation methods produce short-lived results in adult ruminants and currently there is no effective method. Thus, there is an emerging trend towards studying host-microbial interaction in the pre-ruminant rumen to identify best window of opportunity for long-term manipulation of the rumen fermentation. Based on current knowledge, some studies have been able to obtain fairly persisting changes in the rumen microbial composition and fermentation, following interventions. Nevertheless, the impact of these early interventions on adult production performance and phenotypes are still need to be understood. This may also allow identifying the early rumen microbial markers that link to host phenotypes and they can be used in animal selection process in future.
